# Supplementary material for: Steering the Properties of MoOx Hole Transporting Layers in OPVs and OLEDs: Interface Morphology vs. Electronic Structure
Source: Materials (Basel). 2017 Jan 30;10(2):123. doi: 10.3390/ma10020123 (PMC5459185; doi:10.3390/ma10020123)
Supplement: Supplementary file 1 [file materials-10-00123-s001.pdf]

# Supplementary Materials: Steering the Properties of $MoO_x$ Hole Transporting Layers in OPVs and OLEDs: Interface Morphology vs. Electronic Structure

**Wouter Marchal, Inge Verboven, Jurgen Kesters, Boaz Moeremans, Christopher De Dobbelaere, Gilles Bonneux, Ken Elen, Bert Conings, Wouter Maes, Hans Gerd Boyen, Wim Deferme, Marlies Van Bael and An Hardy**

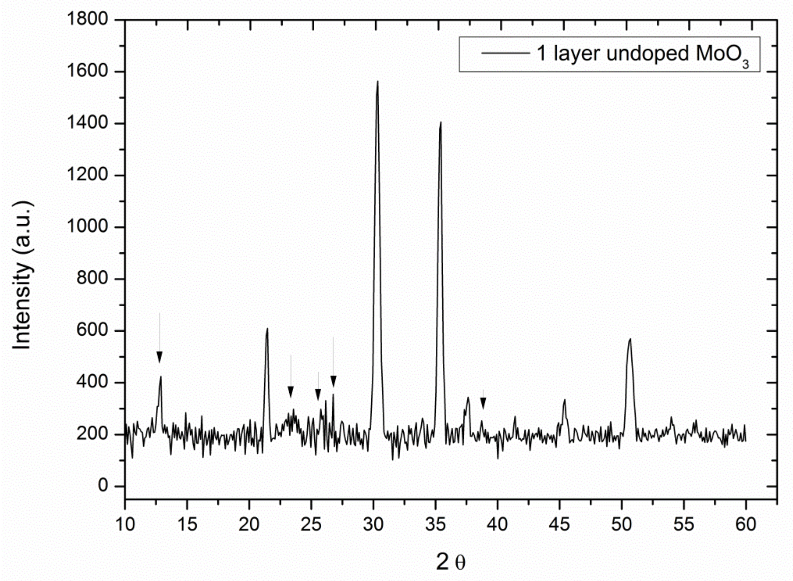


**Figure S1**: diffractogram of a single $MoO_x$ layer in which arrows indicate the observable crystallographic peaks corresponding to o-$MoO_3$. Although signals corresponding to o-$MoO_3$ can already be assigned, the main contribution to the diffractogram is clearly from the underlying indium tin oxide (ITO) substrate (Figure S8).

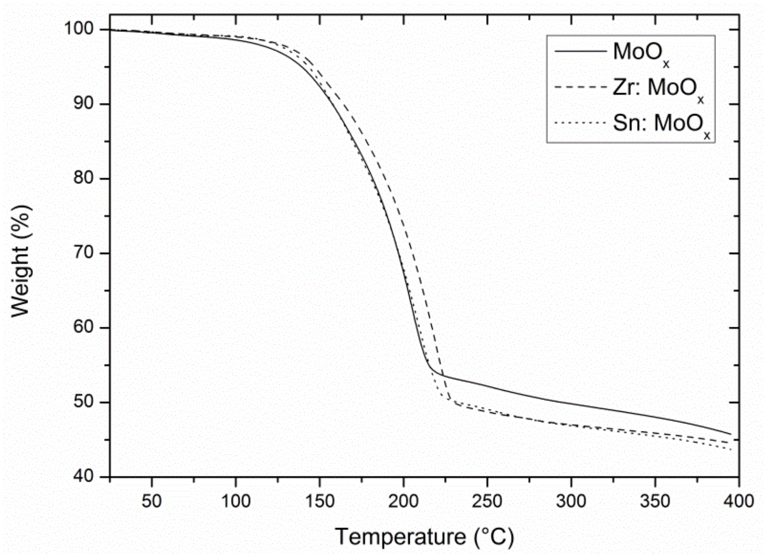


**Figure S2:** Thermogravimetric analysis of the dried $MoO_x$ precursor described in the experimental section and the 0.5 % Sn- and Zr-containing modifications. The drying step was executed on a 80 °C hotplate, and the obtained powder was grinded and sieved (mesh 63 μm). A full explanation on the decomposition process can be found in Reference [30]. The fact that a slightly increased residual mass is found for the pure

$MoO_x$ precursor can be justified by the fact that $Zr(acac)_4$ and $Sn(acac)_2Cl_2$ were added in the multi-metal precursor system, probably causing a slight increase in mass loss because of acetylacetonate decomposition.

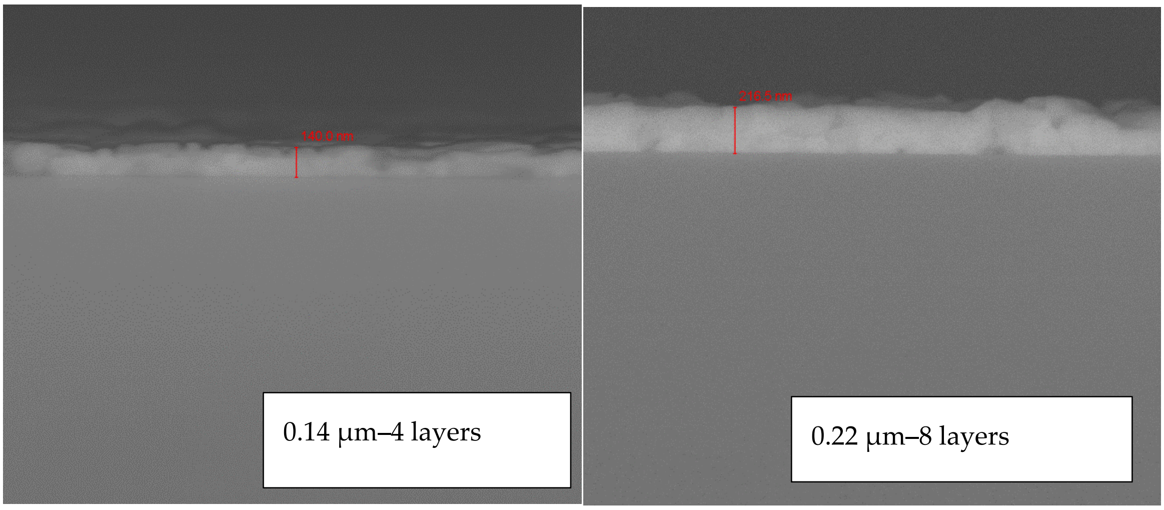


**Figure S3:** Cross-section SEM image of four spin-coated $MoO_x$ layers (left) and eight spin-coated $MoO_x$ layers. Assuming a continuous and proportional growth of the thickness for each layer, the thickness of a single layer is estimated to be around 30 nm. These results and images were previously published in Reference [30] (supporting information Figure S7), but are presented again because of their relevance for this work.

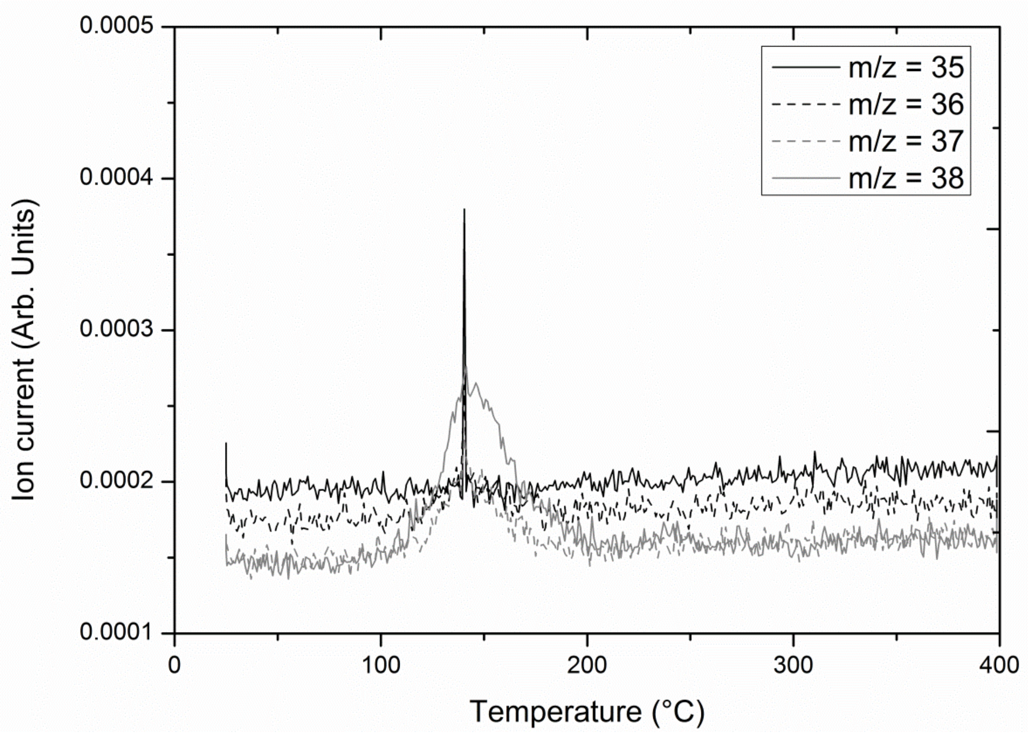


**Figure S4:** TG-MS of *m*/*z* values typically related to Cl and HCl ions in a quadrupole analyzer. Although a clear increase could be observed for all *m*/*z* values, the associated ion currents are very low compared to other decomposition products such as $CO_2$, $NO_2$, and $H_2O$.

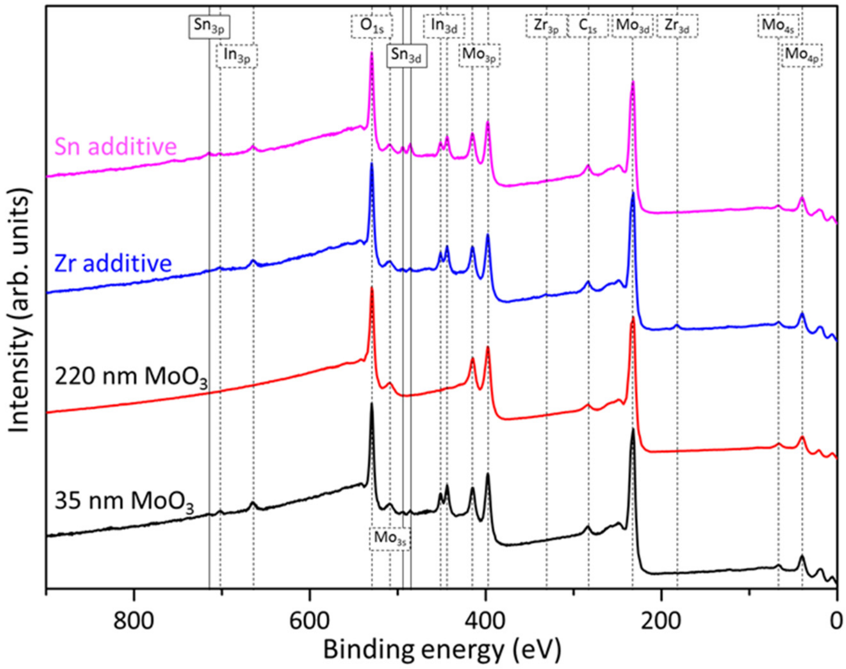


**Figure S5:** Surveys of 35 nm unmodified (no additives), Zr-containing, and Sn-containing $MoO_x$ layers, including a survey of the thick 220 nm sample. Thicknesses are determined via cross-section SEM (X-SEM) as indicated in Figure S3.

**Unmodified $MoO_x$**

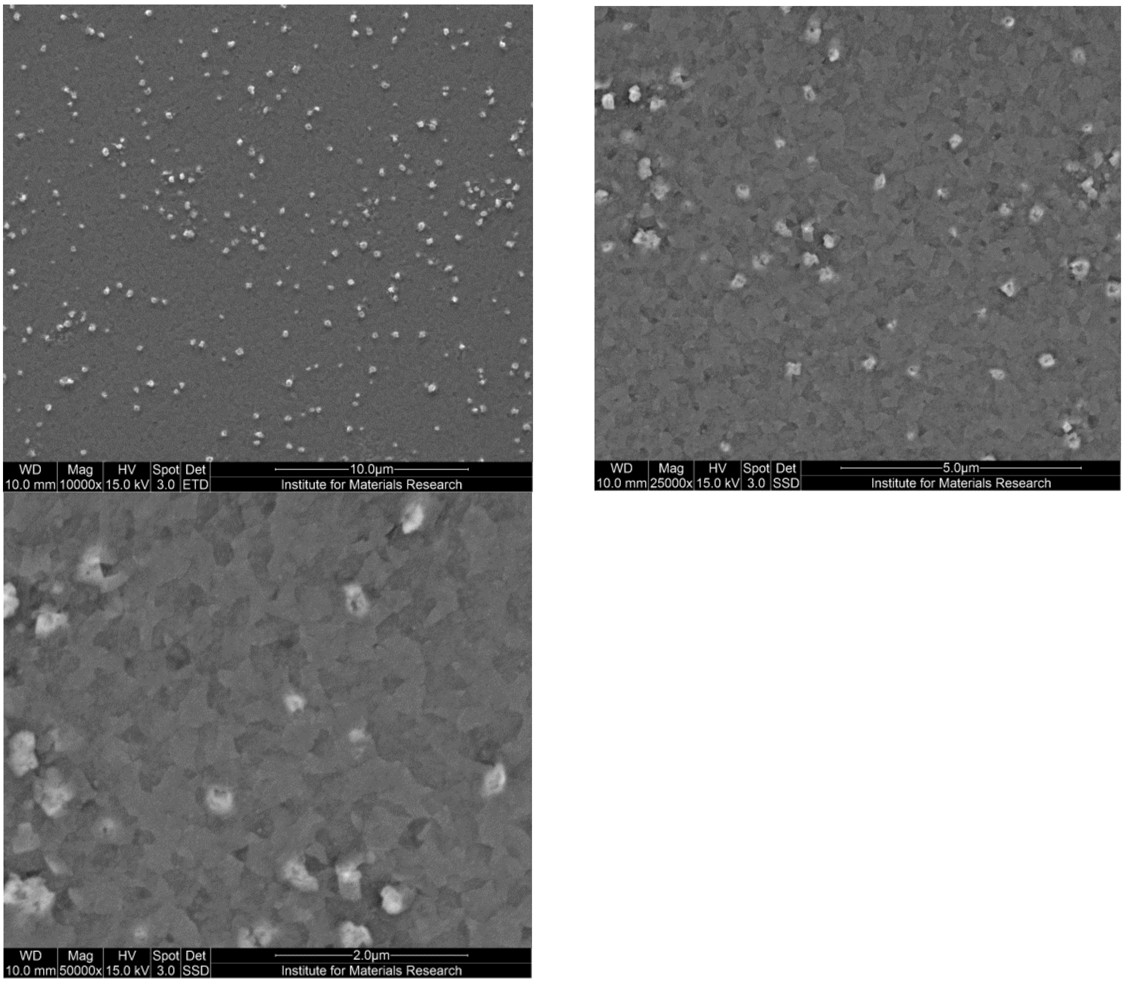

**0.5% Zr-containing $MoO_x$**

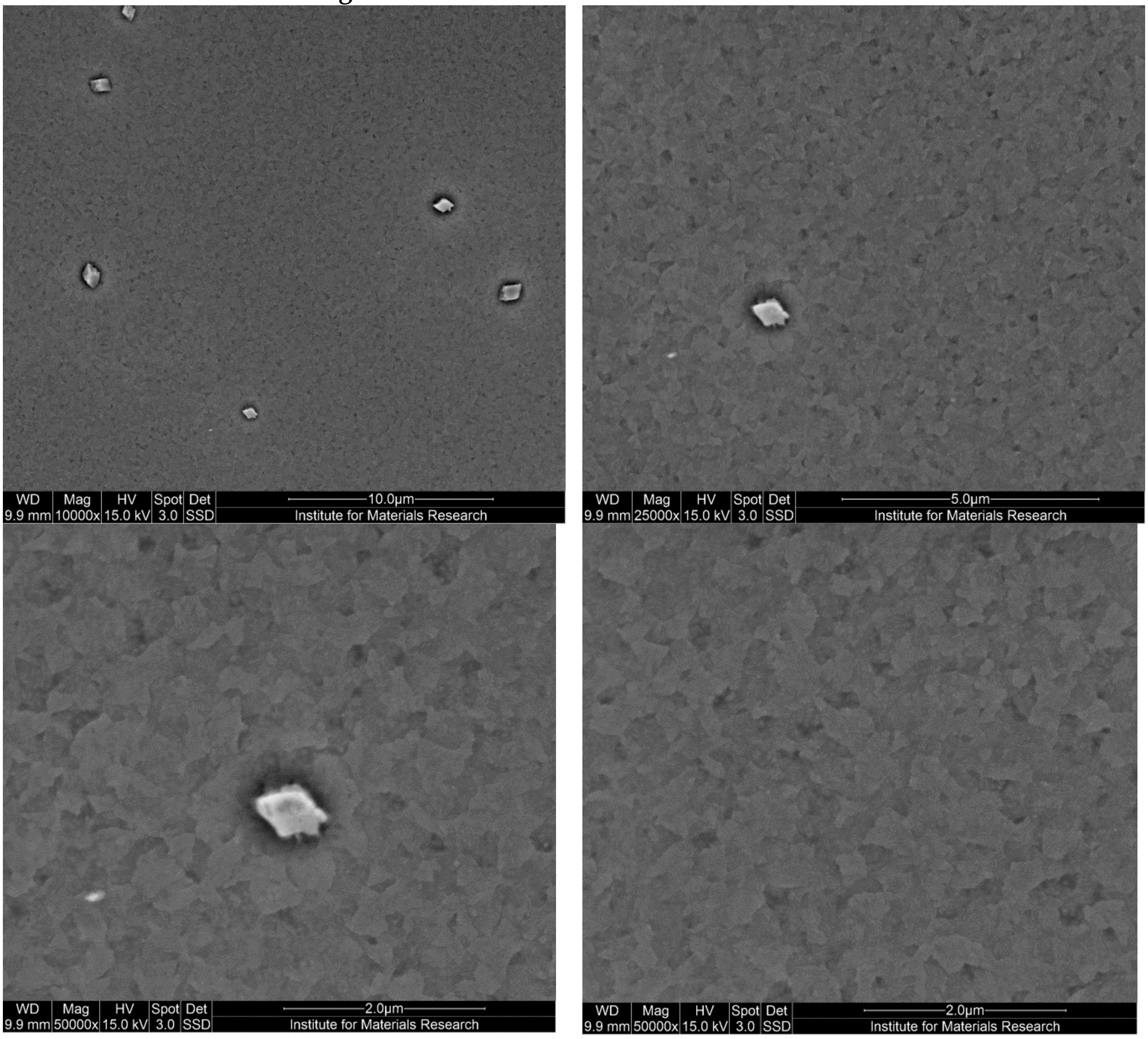


**0.5% Sn-containing $MoO_x$**

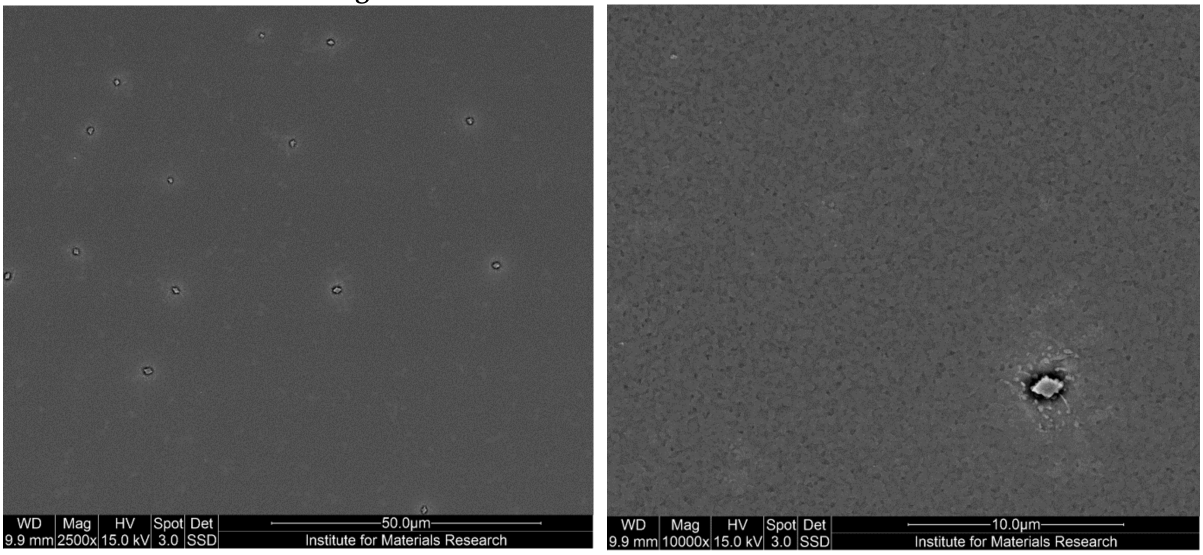

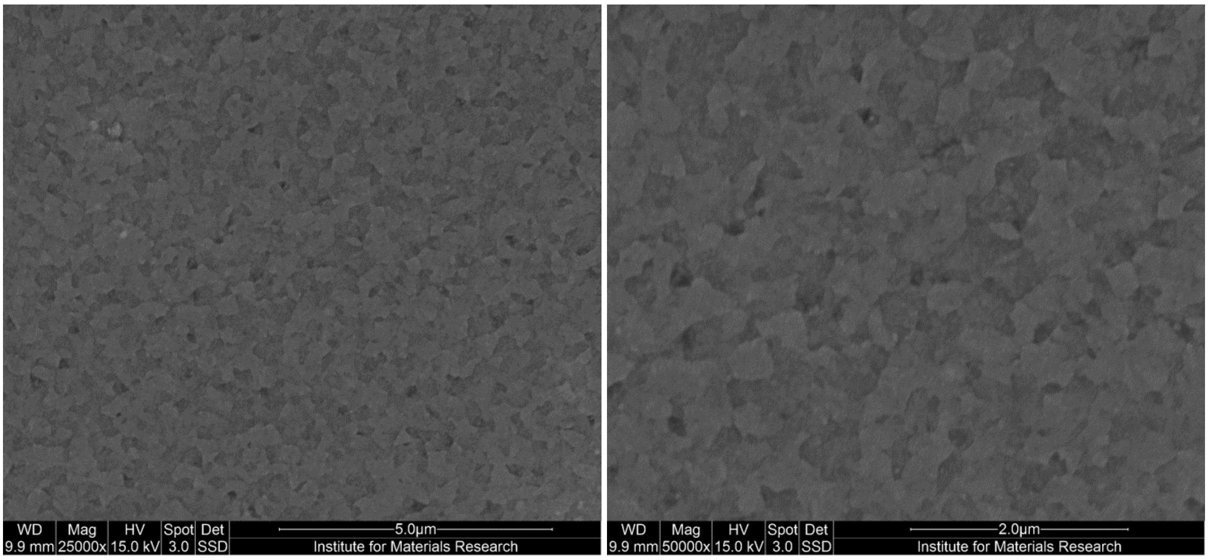


**Figure S6:** SEM images of spin-coated Sn- and Zr-doped and unmodified $MoO_x$ layers thermally treated according to the experimental procedure. Different spots and magnifications (10000x, 25000x, and 50000x, and 2500x for Sn-doped only illustrating the presence of crystallites) are shown to illustrate the systematic change of the morphology.

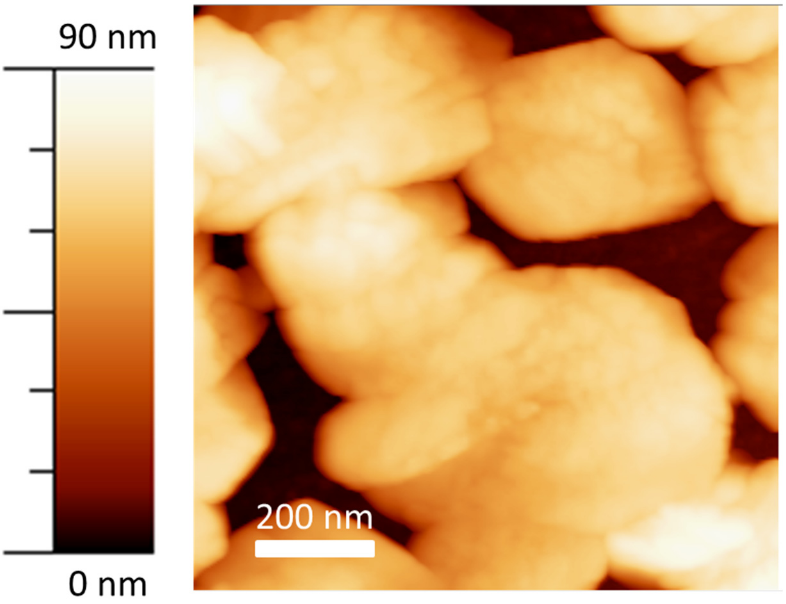


**Figure S7:** Zoomed-in atomic force microscopy (AFM) image illustrating the ununiformity of the deposition near crystalline features. The uncovered parts near the crystallites ccan explain the occurance of the indium signal of the underlying ITO substrate in X-ray photoelectron spectroscopy (XPS).

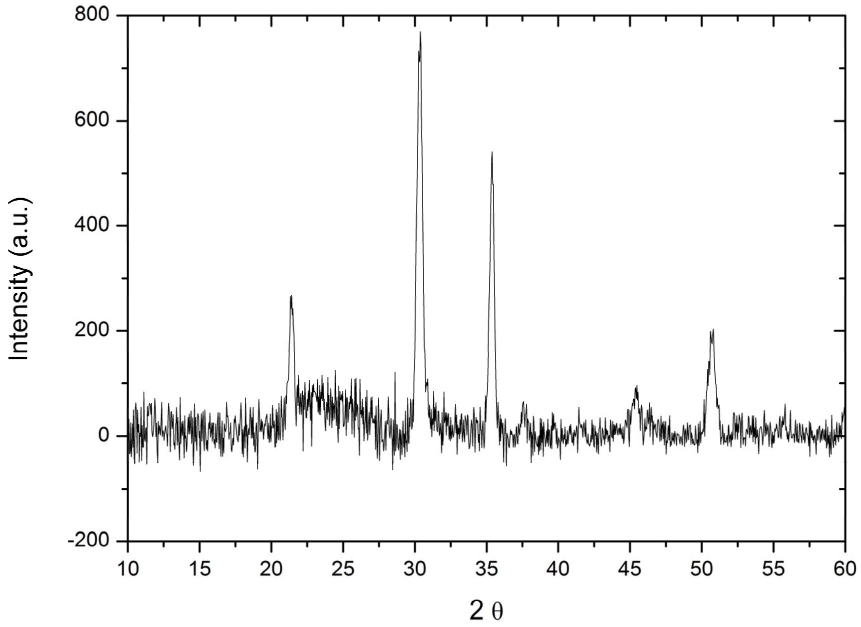


**Figure S8.** diffractogram of ITO background (substrate).

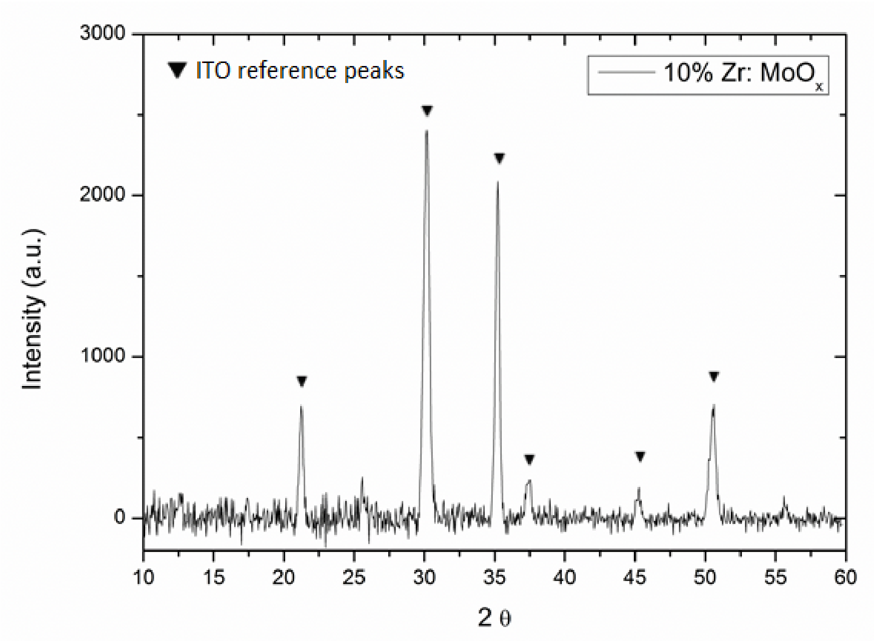


Figure S9: Diffractofram of four subsequently deposited $MoO_x$ layers from a precursor containing 10% Zr. The absence of signals pointing to o-$MoO_3$ or $NH_3(MoO_3)_3$ proves that a higher additive content is also able to act on the nucleation and growth kinetics.

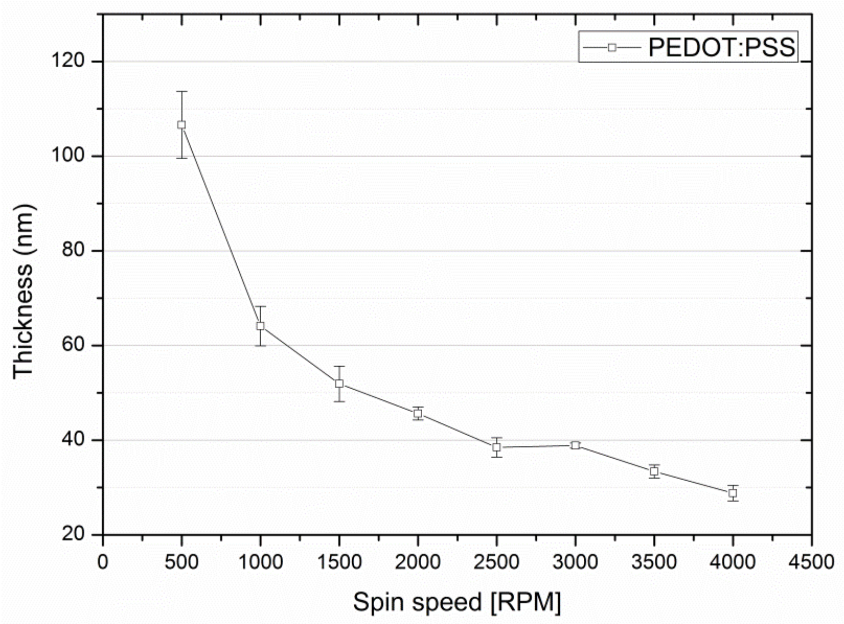


**Figure S10:** PEDOT:PSS thickness determined via profilometry as a function of the spin speed parameter during spin coating. For 35 nm layers, 3500 rpm was used to deposit PEDOT:PSS layers with a thickness comparable to the processed $MoO_x$ layers.

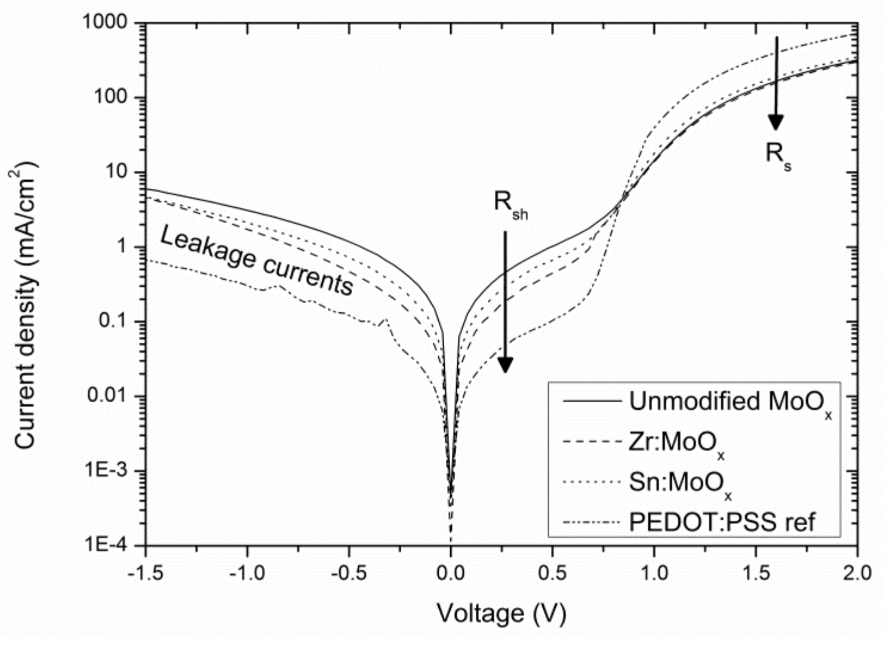


**Figure S11:** Average dark J–V plots of the unmodified $MoO_x$-, Zr:$MoO_x$-, and Sn:$MoO_x$-containing organic photovoltaic (OPV) devices (eight devices each) illustrating higher leakage curents for the $MoO_x$ layers compared to the additive-modified layers and the reference device incorporating PEDOT:PSS. Moreover, a clear trend in the shunt resistance is demonstrated. The PEDOT:PSS layers have a considerably lower series resistance, partially explaining the superior fill factor.

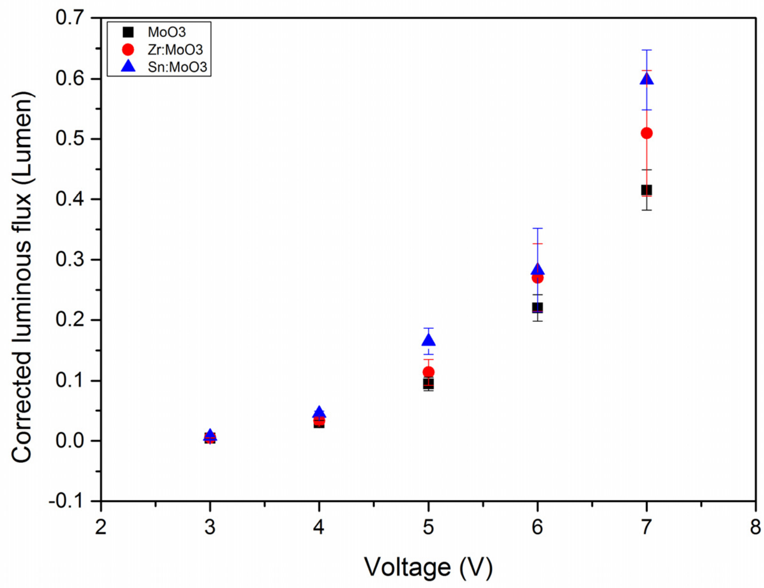


**Figure S12:** Organic light emitting diode (OLED) results from the least succesful badge in terms of luminous flux. Deviations in absolute values are attributed to the lack of atmosphere control in the glovebox because of other activities before the layers were processed and other influencing parameters such as (limited) oxidation of the calcium layer. The yield of functional devices was approximately 60%.

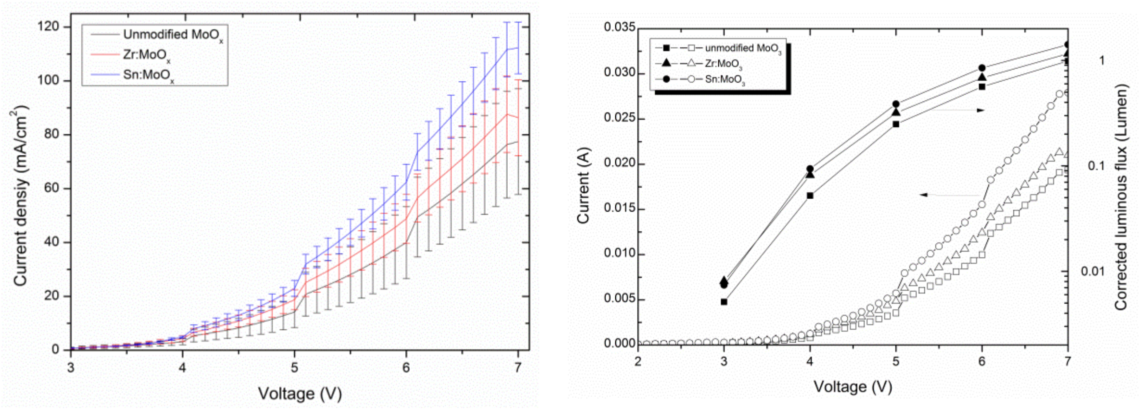


**Figure S13:** J–V curves corresponding to the OLEDs shown in Figure 8 and Figure 9 (left side). The Sn-modified $MoO_x$ layer exbibits the highest current density for all voltages. The current values where measured and converted to current density using the surface of the OLEDs (5 mm × 5 mm). The absolute currents and luminous flux values are represented together in the I–V–L curve on the right side.

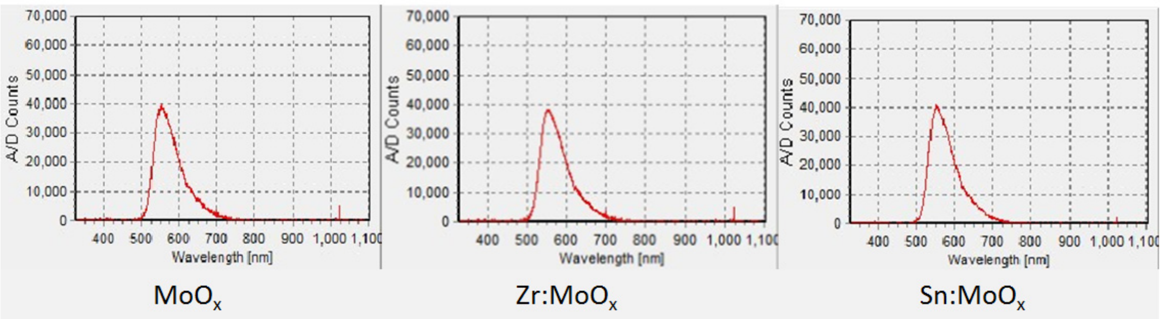


**Figure S14:** Print screens of the emission spectra during OLED chacaterization. The results indicate a similar emission spectrum the devices incorparating the unmodified $MoO_x$ and the Zr- or Sn-containing $MoO_x$ hole transporting layer (HTL). The maximum intesity was located at approximately 550 nm.

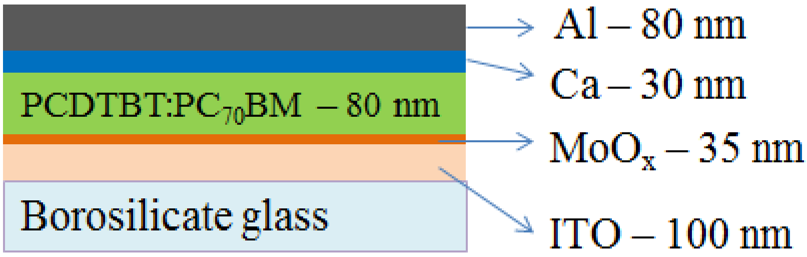


**Figure S15:** OPV divice stack showing the employed materials and layer thicknesses. PCDTBT:$PC_{70}$BM: poly[[9-(1-octylnonyl)-9*H*-carbazole-2,7-diyl]-2,5-thiophenediyl -2,1,3-benzothiadiazole-4,7-diyl-2,5-thiophenediyl]:[6,6]-phenyl-$C_{70}$-butyric acid methyl ester.
